# Supplementary material for: A Statistical Porosity Characterization Approach of Carbon-Fiber-Reinforced Polymer Material Using Optical Microscopy and Neural Network
Source: Materials (Basel). 2022 Sep 21;15(19):6540. doi: 10.3390/ma15196540 (PMC9572467; doi:10.3390/ma15196540)
Supplement: Supplementary file 1 [file materials-15-06540-s001.zip › materials-1902929_SupplementaryMaterial-1.pdf]

```python
# Code Created Sept-2021
# Author: saraeli@kth.se
# -------------------------------------------
'''
Code developed by Sara Eliasson (saraeli@kth.se) - September 2021
Published in MDPI Materials (materials-1902929)
-------------------------------------
Script for training a U-Net for phase characterization of CFRP micrographs.
'''
# -------------------------------------------
# IMPORTS
# -------------------------------------------
import tensorflow as tf
from tensorflow.keras.layers import Conv2D, BatchNormalization, Activation, MaxPool2D,
Conv2DTranspose, Concatenate, Input, CenterCrop
from tensorflow.keras.models import Model
from tensorflow.keras.preprocessing.image import ImageDataGenerator
from sklearn.model_selection import train_test_split
from tensorflow.keras.callbacks import ModelCheckpoint, ReduceLROnPlateau, EarlyStopping,
TensorBoard, CSVLogger
from tensorflow.keras.utils import to_categorical
from tensorflow import image
from tensorflow.image import resize_with_crop_or_pad
import os
import cv2
import matplotlib.pyplot as plt
import numpy as np
from PIL import Image
from random import shuffle
from tqdm import tqdm
from sklearn.utils import class_weight
from sklearn.preprocessing import LabelEncoder

# -------------------------------------------

## =====================================================================================
#
#                                        CLASSES
#
## =====================================================================================
# The U-Net is written as a class using the TensorFlow subclassing Model class.
class CNN_UNet(Model):
    '''
    CNN - Convolutional Neural Network
    The structure of the CNN follows the U-Net architechture presented by Ronneberger 2015.
    '''

    # In TensorFlow, if subclassing Model class: you should define your
    # layers in __init__ and you should implement
    # the model's forward pass in call.

    def __init__(self, input_dim, num_layers, num_classes, batch_size):
        super(CNN_UNet, self).__init__()
        # Define the input variables as methods, for example
        self.input_dim = input_dim
        self.batch_size = batch_size
        self.num_layers = num_layers
        self.num_classes = num_classes

        # VARIABLES FOR THE U-Net:
        NUM_FILTER1 = 64
        NUM_FILTER2 = 128
        NUM_FILTER3 = 256
        NUM_FILTER4 = 512
        NUM_FILTER5 = 1024

        KERNEL_SIZE = 3

        # No padding is used for the U-Net.
        PADDING = 'valid'        # 'valid' = No padding
                                 # 'same' = Padding with zeros

        # Define the convolutional neural network composing elements/layers
        # (check the documentation for the required inputs)
        # The U-Net (Ronneberger 2015), has a contracting path
        # (encoders) and an expansive path (decoders).
```

```
        # ENCODER 1
        self.encoder1_1_Conv = Conv2D(NUM_FILTER1, KERNEL_SIZE, padding=PADDING)
        self.encoder1_1_BatchNorm = BatchNormalization()
        self.encoder1_1_Activation = Activation('relu')
        self.encoder1_2_Conv = Conv2D(NUM_FILTER1, KERNEL_SIZE, padding=PADDING)
        self.encoder1_2_BatchNorm = BatchNormalization()
        self.encoder1_2_Activation = Activation('relu')
        self.encoder1_2_Pooling = MaxPool2D(pool_size=(2,2))
        self.encoder1_crop4concatenation = CenterCrop(392,392)

        # ENCODER 2
        self.encoder2_1_Conv = Conv2D(NUM_FILTER2, KERNEL_SIZE, padding=PADDING)
        self.encoder2_1_BatchNorm = BatchNormalization()
        self.encoder2_1_Activation = Activation('relu')
        self.encoder2_2_Conv = Conv2D(NUM_FILTER2, KERNEL_SIZE, padding=PADDING)
        self.encoder2_2_BatchNorm = BatchNormalization()
        self.encoder2_2_Activation = Activation('relu')
        self.encoder2_2_Pooling = MaxPool2D(pool_size=(2, 2))
        self.encoder2_crop4concatenation = CenterCrop(200,200)

        # ENCODER 3
        self.encoder3_1_Conv = Conv2D(NUM_FILTER3, KERNEL_SIZE, padding=PADDING)
        self.encoder3_1_BatchNorm = BatchNormalization()
        self.encoder3_1_Activation = Activation('relu')
        self.encoder3_2_Conv = Conv2D(NUM_FILTER3, KERNEL_SIZE, padding=PADDING)
        self.encoder3_2_BatchNorm = BatchNormalization()
        self.encoder3_2_Activation = Activation('relu')
        self.encoder3_2_Pooling = MaxPool2D(pool_size=(2, 2))
        self.encoder3_crop4concatenation = CenterCrop(104,104)

        # ENCODER 4
        self.encoder4_1_Conv = Conv2D(NUM_FILTER4, KERNEL_SIZE, padding=PADDING)
        self.encoder4_1_BatchNorm = BatchNormalization()
        self.encoder4_1_Activation = Activation('relu')
        self.encoder4_2_Conv = Conv2D(NUM_FILTER4, KERNEL_SIZE, padding=PADDING)
        self.encoder4_2_BatchNorm = BatchNormalization()
        self.encoder4_2_Activation = Activation('relu')
        self.encoder4_2_Pooling = MaxPool2D(pool_size=(2, 2))
        self.encoder4_crop4concatenation = CenterCrop(56,56)

        # BRIDGE
        self.bridge_1_Conv = Conv2D(NUM_FILTER5, KERNEL_SIZE, padding=PADDING)
        self.bridge_1_BatchNorm = BatchNormalization()
        self.bridge_1_Activation = Activation('relu')
        self.bridge_2_Conv = Conv2D(NUM_FILTER5, KERNEL_SIZE, padding=PADDING)
        self.bridge_2_BatchNorm = BatchNormalization()
        self.bridge_2_Activation = Activation('relu')

        # DECODER 1
        self.decoder1_1_ConvTrans = Conv2DTranspose(NUM_FILTER4, (2,2), strides=2,
padding=PADDING)
        self.decoder1_1_Concatenate = Concatenate() # Retaining spatial information (features
from encoder added to decoder)
        self.decoder1_2_Conv = Conv2D(NUM_FILTER4, KERNEL_SIZE, padding=PADDING)
        self.decoder1_2_BatchNorm = BatchNormalization()
        self.decoder1_2_Activation = Activation('relu')
        self.decoder1_3_Conv = Conv2D(NUM_FILTER4, KERNEL_SIZE, padding=PADDING)
        self.decoder1_3_BatchNorm = BatchNormalization()
        self.decoder1_3_Activation = Activation('relu')

        # DECODER 2
        self.decoder2_1_ConvTrans = Conv2DTranspose(NUM_FILTER3, (2, 2), strides=2,
padding=PADDING)
        self.decoder2_1_Concatenate = Concatenate()
        self.decoder2_2_Conv = Conv2D(NUM_FILTER3, KERNEL_SIZE, padding=PADDING)
        self.decoder2_2_BatchNorm = BatchNormalization()
        self.decoder2_2_Activation = Activation('relu')
        self.decoder2_3_Conv = Conv2D(NUM_FILTER3, KERNEL_SIZE, padding=PADDING)
        self.decoder2_3_BatchNorm = BatchNormalization()
        self.decoder2_3_Activation = Activation('relu')

        # DECODER 3
        self.decoder3_1_ConvTrans = Conv2DTranspose(NUM_FILTER2, (2, 2), strides=2,
padding=PADDING)
        self.decoder3_1_Concatenate = Concatenate()
        self.decoder3_2_Conv = Conv2D(NUM_FILTER2, KERNEL_SIZE, padding=PADDING)
        self.decoder3_2_BatchNorm = BatchNormalization()
```

```
        self.decoder3_2_Activation = Activation('relu')
        self.decoder3_3_Conv = Conv2D(NUM_FILTER2, KERNEL_SIZE, padding=PADDING)
        self.decoder3_3_BatchNorm = BatchNormalization()
        self.decoder3_3_Activation = Activation('relu')

        # DECODER 4
        self.decoder4_1_ConvTrans = Conv2DTranspose(NUM_FILTER1, (2, 2), strides=2,
padding=PADDING)
        self.decoder4_1_Concatenate = Concatenate()
        self.decoder4_1_Conv = Conv2D(NUM_FILTER1, KERNEL_SIZE, padding=PADDING)
        self.decoder4_2_BatchNorm = BatchNormalization()
        self.decoder4_2_Activation = Activation('relu')
        self.decoder4_3_Conv = Conv2D(NUM_FILTER1, KERNEL_SIZE, padding=PADDING)
        self.decoder4_3_BatchNorm = BatchNormalization()
        self.decoder4_3_Activation = Activation('relu')

        # OUTPUT LAYER
        # For this multiclass segmentation we use the number of classes as
        # the output feature channel with softmax activation function.
        self.final_output = Conv2D(num_classes, (1,1), padding=PADDING, activation='softmax')

    # Define the forward pass
    def call(self, x):
        # The input is fed to the recurrent layer
        # ENCODER 1
        x1 = self.encoder1_1_Conv(x)
        x1 = self.encoder1_1_BatchNorm(x1)
        x1 = self.encoder1_1_Activation(x1)
        x1 = self.encoder1_2_Conv(x1)
        x1 = self.encoder1_2_BatchNorm(x1)
        x1 = self.encoder1_2_Activation(x1)
        p1 = self.encoder1_2_Pooling(x1)
        x1 = self.encoder1_crop4concatenation(x1)   # This is done if PADDING = 'valid'

        # ENCODER 2
        x2 = self.encoder2_1_Conv(p1)
        x2 = self.encoder2_1_BatchNorm(x2)
        x2 = self.encoder2_1_Activation(x2)
        x2 = self.encoder2_2_Conv(x2)
        x2 = self.encoder2_2_BatchNorm(x2)
        x2 = self.encoder2_2_Activation(x2)
        p2 = self.encoder2_2_Pooling(x2)
        x2 = self.encoder2_crop4concatenation(x2)   # This is done if PADDING = 'valid'

        # ENCODER 3
        x3 = self.encoder3_1_Conv(p2)
        x3 = self.encoder3_1_BatchNorm(x3)
        x3 = self.encoder3_1_Activation(x3)
        x3 = self.encoder3_2_Conv(x3)
        x3 = self.encoder3_2_BatchNorm(x3)
        x3 = self.encoder3_2_Activation(x3)
        p3 = self.encoder3_2_Pooling(x3)
        x3 = self.encoder3_crop4concatenation(x3)   # This is done if PADDING = 'valid'

        # ENCODER 4
        x4 = self.encoder4_1_Conv(p3)
        x4 = self.encoder4_1_BatchNorm(x4)
        x4 = self.encoder4_1_Activation(x4)
        x4 = self.encoder4_2_Conv(x4)
        x4 = self.encoder4_2_BatchNorm(x4)
        x4 = self.encoder4_2_Activation(x4)
        p4 = self.encoder4_2_Pooling(x4)
        x4 = self.encoder4_crop4concatenation(x4)   # This is done if PADDING = 'valid'

        # BRIDGE
        x5 = self.bridge_1_Conv(p4)
        x5 = self.bridge_1_BatchNorm(x5)
        x5 = self.bridge_1_Activation(x5)
        x5 = self.bridge_2_Conv(x5)
        x5 = self.bridge_2_BatchNorm(x5)
        x5 = self.bridge_2_Activation(x5)

        # DECODER 1
        x6 = self.decoder1_1_ConvTrans(x5)
        x6 = self.decoder1_1_Concatenate([x6, x4])
        x6 = self.decoder1_2_Conv(x6)
        x6 = self.decoder1_2_BatchNorm(x6)
```

```python
        x6 = self.decoder1_2_Activation(x6)
        x6 = self.decoder1_3_Conv(x6)
        x6 = self.decoder1_3_BatchNorm(x6)
        x6 = self.decoder1_3_Activation(x6)

        # DECODER 2
        x7 = self.decoder2_1_ConvTrans(x6)
        x7 = self.decoder2_1_Concatenate([x7, x3])
        x7 = self.decoder2_2_Conv(x7)
        x7 = self.decoder2_2_BatchNorm(x7)
        x7 = self.decoder2_2_Activation(x7)
        x7 = self.decoder2_3_Conv(x7)
        x7 = self.decoder2_3_BatchNorm(x7)
        x7 = self.decoder2_3_Activation(x7)

        # DECODER 3
        x8 = self.decoder3_1_ConvTrans(x7)
        x8 = self.decoder3_1_Concatenate([x8, x2])
        x8 = self.decoder3_2_Conv(x8)
        x8 = self.decoder3_2_BatchNorm(x8)
        x8 = self.decoder3_2_Activation(x8)
        x8 = self.decoder3_3_Conv(x8)
        x8 = self.decoder3_3_BatchNorm(x8)
        x8 = self.decoder3_3_Activation(x8)

        # DECODER 4
        x9 = self.decoder4_1_ConvTrans(x8)
        x9 = self.decoder4_1_Concatenate([x9, x1])
        x9 = self.decoder4_1_Conv(x9)
        x9 = self.decoder4_2_BatchNorm(x9)
        x9 = self.decoder4_2_Activation(x9)
        x9 = self.decoder4_3_Conv(x9)
        x9 = self.decoder4_3_BatchNorm(x9)
        x9 = self.decoder4_3_Activation(x9)

        # OUTPUT LAYER
        finalOutput = self.final_output(x9)

        return finalOutput

## ======================================================================================
#
#                                   MAIN FUNCTION
#
## ======================================================================================
def main():
    '''
    The main function controls the training of the U-Net.
    The U-Net is implemented in TensorFlow and used to extract different phases
    from CFRP micrographs.
    --------------------------------
    There are five steps in the implementation:
    1. Define the model.
    2. Compile the model.
    3. Fit the model.
    4. Evaluate the model.
    5. Make predictions.
    --------------------------------
    Segmentation of CFRP
    3 Classes are characterized
    Pixel Value     -   Object   -   New Pixel Value
        1           -   Fiber    -       0
        2           -   Matrix   -       1
        3           -   Void     -       2
    '''
    # -------------------------------------------
    #                   LOAD DATA
    # -------------------------------------------
    # Load and prepare the data for the training, testing and validation.

    # -------------------------------------------
    # We are working with grayscale images, input size (572,572,1)

    # -------------------------------------------
```

```
    # The script sorts the files accordingly.
    # The prerequisites are that your masked images are in one folder and
    # your originals in another and that they have matching names.
    data_path = 'X:/yourdatapath/'
    train_org_folder = 'Originals/' # Folder in data_path were the original images for
training are kept.
    train_mask_folder = 'Masked/' # Folder in data_path were the masked images for training
are kept.

    # Folders for validation images
    valid_org_folder = 'Valid_Originals/'   # Also used for testing the network
    valid_mask_folder = 'Valid_Masked/'     # Also used for testing the network
    # ------------------------------------
    # Path for results
    save_path = 'X:/yourdatapath_save/'

    # ------------------------------------
    log_name = 'training_data.log'   # Name for file to save the training data
    save_log = 'X:/yourdatapath_savelog/' + log_name

    # Save the U-Net model (two models are saved,
    # the best that is updated during training, and the final)
    modelSave1 = 'X:/yourdatapath_saveModel/Unet.tf'  # saving the best model
    modelSave2 = 'X:/yourdatapath_saveModel/Unet_final.tf'  # saving the final model

    # If there is a separate folder for the validation images that
    # shall be unseen by the network...
    valid_path = 'X:/yourdatapath_validation/

    # ------------------------------------------
    # Sorting the images
    # ------------------------------------------
    # The code sort the images. If there are a lot of images and they are presorted,
    # this part can be skipped

    # The complete path to the training/testing/validation files
    train_org_path = os.path.join(data_path, train_org_folder)
    train_mask_path = os.path.join(data_path, train_mask_folder)
    valid_org_path = os.path.join(valid_path, valid_org_folder)
    valid_mask_path = os.path.join(valid_path, valid_mask_folder)

    train_org_images = os.listdir(train_org_path)
    train_mask_images = os.listdir(train_mask_path)
    valid_org_images = os.listdir(valid_org_path)
    valid_mask_images = os.listdir(valid_mask_path)

    train_org = train_org_images
    train_mask = train_mask_images
    test_org = valid_org_images
    test_mask = valid_mask_images

    valid_org = valid_org_images
    valid_mask = valid_mask_images

    # ------------------------------------------
    # An option is to have all available images in one folder and sort them from there.
    # Then this part of the code can be used.

    # Split Data
    # 80%, 15%, 5% (training, testing and validation)
    #train_org, test_org, train_mask, test_mask = train_test_split(train_org_images,
train_mask_images, test_size=0.2, random_state=57)  # 15% for "testing", 80% for training
    #test_org, valid_org, test_mask, valid_mask = train_test_split(test_org, test_mask,
test_size=0.25, random_state=57) # Split the remaining data in testing and validation
    # ------------------------------------------
    # ------------------------------------------
    print(train_org)
    print(train_mask)
    print(test_org)
    print(test_mask)
    print(valid_org)
    print(valid_mask)
    print('.............................')
    print('Number of training images: ', len(train_org))
    print('Number of testing images: ', len(test_org))
    print('Number of validation images: ', len(valid_org))
```

```python
    # ------------------------------------------
    # Now we have structured the data and need to sort them in given folders
    folders = ['train_images', 'train_masks', 'test_images', 'test_masks', 'valid_images',
'valid_masks']
    for folder in folders:
        existing = os.listdir(data_path)
        if folder not in existing:
            os.makedirs(data_path + folder)

    image_folders = [(train_org, 'train_images'), (test_org, 'test_images'),
                     (valid_org, 'valid_images')]

    mask_folders = [(train_mask, 'train_masks'), (test_mask, 'test_masks'),
                    (valid_mask, 'valid_masks')]

    # Move images to right folder, originals and masks.
    for folder in image_folders:
        images = folder[0]
        folderName = folder[1]
        for image in images:
            if folderName == 'valid_images':
                img = Image.open(valid_org_path + image)
            elif folderName == 'test_images':
                img = Image.open(valid_org_path + image)
            else:
                img = Image.open(train_org_path + image)
            img.save(data_path + folderName + '/' + image)

    for folder in mask_folders:
        images = folder[0]
        folderName = folder[1]
        for image in images:
            if folderName == 'valid_masks':
                img = Image.open(valid_mask_path + image)
            elif folderName == 'test_masks':
                img = Image.open(valid_mask_path + image)
            else:
                img = Image.open(train_mask_path + image)
            img.save(data_path + folderName + '/' + image)

    # ------------------------------------------
    # SET PARAMETERS
    # ------------------------------------------
    # Image size:
    #img_W = 512    # With padding
    #img_H = 512    # With padding
    img_W = 572     # Without padding
    img_H = 572     # Without padding

    batchSize = 20
    bufferSize = 1000  # How many images do you have as input? Make sure this variable is
larger.
    numberClasses = 3   # void, matrix, fiber

    # ------------------------------------------
    # FIX DATA SO THAT IT IS SUITED FOR TRAINING
    # ------------------------------------------
    # ------------------------------------------
    # Creating the datasets from the original images
    print('..............................')
    print('Creating the datasets ...')
    train_image_ds = []
    test_image_ds = []
    valid_image_ds = []
    loopNbr = 0
    for folder in image_folders:
        images = folder[0]
        folderName = folder[1]
        for image in images:
            image_path = data_path+folderName+'/'+image
            img = cv2.imread(image_path, cv2.IMREAD_GRAYSCALE)
            # Shape of the image is here 512x512 (with padding)
            # or 572x572 (without padding), read as a grayscale image
            # We want it to be (x,x,1) before moving on.
            #img = cv2.resize(img, (img_H, img_W))     # If we need to resize the image
            img = img.reshape(img_H, img_W, 1)
```

```
        img = img.astype(np.float32)
        img = img/255.0 # Normalize the values

        if loopNbr == 0:
            train_image_ds.append(np.array(img))
        if loopNbr == 1:
            test_image_ds.append(np.array(img))
        if loopNbr == 2:
            valid_image_ds.append(np.array(img))
    loopNbr = loopNbr + 1

# Convert to numpy arrays
train_image_ds = np.array(train_image_ds)
test_image_ds = np.array(test_image_ds)
valid_image_ds = np.array(valid_image_ds)
print('............................')
print('Shape of numpy array with training, testing and validation images')
print(train_image_ds.shape, test_image_ds.shape, valid_image_ds.shape)

# Creating the datasets from the masked images
# NOTE: The masked images need to be in the shape (img_W,img_H,numberClasses)
# for the learning to work.
train_mask_ds = []
test_mask_ds = []
valid_mask_ds = []
weight_vector = []
loopNbr = 0
for folder in mask_folders:
    images = folder[0]
    folderName = folder[1]
    for image in images:
        image_path = data_path + folderName + '/' + image
        img = cv2.imread(image_path, cv2.IMREAD_GRAYSCALE)
        # PLOT THE IMAGES
        #plt.figure()
        #plt.imshow(img)
        #plt.show()

        # With current settings our mask images has pixel values
        # 0 - void, 1 - matrix, 2 - fiber
        # This is good so we don't change any pixel values
        #img = cv2.resize(img, (img_H, img_W))      # If we need to resize the image
        img = img.astype(np.int32)  # Turn into np array
        weight_vector.append(img)   # adds all masks to one vector

        # Shape of the image is now (img_w,img_H)
        # We want it to be (img_w, img_H,3) before moving on.
        # Creates the correct layers for the masks, one layer for each output class.
        # [1,0,0] - void, [0,1,0] - matrix, [0,0,1] - fiber
        img = tf.keras.utils.to_categorical(img, num_classes=numberClasses, dtype='int32')

        if loopNbr == 0:
            train_mask_ds.append(img)
        if loopNbr == 1:
            test_mask_ds.append(img)
        if loopNbr == 2:
            valid_mask_ds.append(img)
    loopNbr = loopNbr + 1

# Convert to numpy arrays
train_mask_ds = np.array(train_mask_ds)
test_mask_ds = np.array(test_mask_ds)
valid_mask_ds = np.array(valid_mask_ds)
weight_vector = np.array(weight_vector)
print('............................')
print('Shape of numpy array with training, testing and validation images')
print(train_mask_ds.shape, test_mask_ds.shape, valid_mask_ds.shape)

# ------------------------------------
# ANALYSING THE WEIGHTS OF THE CLASSES
# Use sample_weights
# The shape of the sample weights shall be: ()
# The sample weight describes the weight for each training sample,
# used for weighting in the loss function during training.
```

```python
    # Checking for unbalanced data
    print('..............................')
    print('Unique values in the weight vector: ', np.unique(weight_vector))
    weight_classes = np.unique(weight_vector)
    weight_classes.astype(np.int32)
    # Flatten out the data to one dimensional
    weight_vector_reshaped = weight_vector.reshape(-1,1)
    weight_vector_reshaped = np.asarray(weight_vector_reshaped)
    # Encoding to shape - (x,)
    labelencoder = LabelEncoder()
    weight_vector_reshaped = labelencoder.fit_transform(weight_vector_reshaped)
    class_weights = class_weight.compute_class_weight('balanced',
np.unique(weight_vector_reshaped), weight_vector_reshaped)
    # Create a dictionary
    class_weights_dicts = {weight_classes[0]: class_weights[0], weight_classes[1]:
class_weights[1], weight_classes[2]: class_weights[2]}
    print('The class weights are:', class_weights_dicts)

    # -----------------------------------
    print('..............................')
    print('The final training, testing and validation data in numpy arrays: ')
    print('Training dataset: ', train_image_ds.shape, train_mask_ds.shape)
    print('Testing dataset: ', test_image_ds.shape, test_mask_ds.shape)
    print('Validation dataset: ', valid_image_ds.shape, valid_mask_ds.shape)

    # -----------------------------------
    # CREATE TensorFlow DATASET
    print('..............................')
    print('Creating the TensorFlow dataset (.from_tensor_slices) ...')
    train_ds = tf.data.Dataset.from_tensor_slices((train_image_ds, train_mask_ds))
    test_ds = tf.data.Dataset.from_tensor_slices((test_image_ds, test_mask_ds))
    valid_ds = tf.data.Dataset.from_tensor_slices((valid_image_ds, valid_mask_ds))

    print('TensorFlow Datasets are created! ...')

    train_ds = train_ds.shuffle(buffer_size=bufferSize, reshuffle_each_iteration=True)
    train_ds = train_ds.batch(batchSize)
    train_ds = train_ds.repeat()
    train_ds = train_ds.prefetch(5)   # Speeding up...

    test_ds = test_ds.shuffle(buffer_size=bufferSize, reshuffle_each_iteration=True)
    test_ds = test_ds.batch(batchSize)
    test_ds = test_ds.repeat()
    test_ds = test_ds.prefetch(5)  # Speeding up...

    valid_ds = valid_ds.shuffle(buffer_size=bufferSize, reshuffle_each_iteration=True)
    valid_ds = valid_ds.batch(batchSize)
    valid_ds = valid_ds.repeat()
    valid_ds = valid_ds.prefetch(5)  # Speeding up...

    print('..............................')
    print('Summary of the datasets created: ')
    print('Training dataset: ', train_ds)
    print('Testing dataset: ', test_ds)
    print('Validation dataset: ', valid_ds)

    # -------------------------------------------
    #       PARAMETERS FOR THE TRAINING
    # -------------------------------------------
    #inputShape = (512, 512, 1)     # With padding
    inputShape = (572, 572, 1)      # Without padding
    learnRate = 1e-4
    EPOCHS = 200

    # -------------------------------------------
    # 1. Define the Model:
    # -------------------------------------------
    # The model is defined in the class CNN_UNet()
    model = CNN_UNet(inputShape, 3, numberClasses, batchSize)
    # -------------------------------------------

    print('..............................')
    print('The model is defined with ')
    print('Batch size = ', batchSize)
    print('EPOCHS = ', EPOCHS)
```

```
    # ------------------------------------------
    # 2. Compile the Model:
    # ------------------------------------------
    print('.............................')
    print('Compiling model ...')
    # Compile the model and set the loss function and optimizer.
    # The loss should for multiclass be categorical_crossentropy or multicross_entropy.
    model.compile(loss='categorical_crossentropy',
optimizer=tf.keras.optimizers.Adam(learnRate), metrics=['accuracy'])

    train_steps = len(train_org)//batchSize
    test_steps = len(test_org)//batchSize
    print('.............................')
    print('Steps per EPOCH = ', train_steps)

    print('.............................')
    print('Training the model ...')
    print('.............................')

    # ------------------------------------------
    # SETTING CALLBACKS
    mc = ModelCheckpoint(modelSave1, verbose=1, save_best_only=True)
    #mc = ModelCheckpoint('Unet.tf', monitor='val_accuracy', mode='max', verbose=1,
save_best_only=True)

    # Adjusting the learning rate
    rlp = ReduceLROnPlateau(monitor='val_loss', patience=4, factor=0.1, verbose=1, min_lr=1e-
6)

    # Start with keeping it simple, add callbacks when you know everything is working
    #es = EarlyStopping(monitor='val_loss', patience=10, verbose=1,
restore_best_weights=False)

    # Saving the history data from the run
    csv_logger = CSVLogger(save_log, separator=',', append=False)
    #callbacks = [mc, csv_logger]
    callbacks = [mc, csv_logger, rlp]
    #callbacks = [mc, csv_logger, rlp, es]

    # ------------------------------------------
    # 3. Fit the model.
    # ------------------------------------------
    # Starting the training of the model
    history = model.fit(train_ds, steps_per_epoch=train_steps, validation_data=test_ds,
validation_steps=test_steps,
              epochs=EPOCHS, callbacks=callbacks)

    # If your data is unequally weighted an option is to
    # take the weights of the classes into consideration.
    #history = model.fit(train_ds, steps_per_epoch=train_steps, validation_data=test_ds,
validation_steps=test_steps,
    #                    epochs=EPOCHS, callbacks=callbacks, class_weight=class_weights_dicts)

    # ------------------------------------------
    # 4. Evaluate the model.
    # ------------------------------------------
    print('.............................')
    print('Listing all data in history ...')
    print(history.history.keys())

    print('.............................')
    print('Training accuracy ...')
    _, train_acc = model.evaluate(train_ds, steps=train_steps, verbose=1)
    _, test_acc = model.evaluate(test_ds, steps=test_steps, verbose=1)
    print('Train: %.3f, Test: %.3f' % (train_acc, test_acc))

    print('.............................')
    print(' Plotting ...')
    # Plot training history
    plt.figure()
    plt.plot(history.history['loss'], 'bo', label='Training Loss')
    plt.plot(history.history['val_loss'], 'b', label='Validation Loss')
    plt.title('Training and Validation Loss')
    plt.legend()
    plt.savefig(save_path+'testplot_loss.png')
    # ------------------------------------------
```

```
    plt.figure()
    plt.plot(history.history['accuracy'], 'bo', label='Training Accuracy')
    plt.plot(history.history['val_accuracy'], 'b', label='Validation Accuracy')
    plt.title('Training and Validation Accuracy')
    plt.legend()
    plt.savefig(save_path + 'testplot_acc.png')
    plt.show()

    # Print summary of the NN model
    print('.............................')
    print('MODEL SUMMARY')
    print('.............................')
    print(model.summary())

    print('.............................')
    print('Saving the model')
    model.save(modelSave2, save_format='tf')

    # -------------------------------------------
    # 5. Make predictions.
    # -------------------------------------------
    # Testing/Validation
    # LOAD THE MODEL
    #model = tf.keras.models.load_model(modelSave1)
    model = tf.keras.models.load_model(modelSave2)
    print('.............................')
    print('Loaded the trained model for validation')

    # -------------------------------------------
    # VALIDATION OF TRAINING
    # File location for validation images
    validation_images = image_folders[2][0] # list with validation images
    validation_mask = mask_folders[2][0]  # list with validation masks
    validation_folder_images = image_folders[2][1] # string with name of folder for validation
images
    validation_folder_masks = mask_folders[2][1] # string with name of folder for validation
masks

    validation_images_path = data_path + validation_folder_images + '/'
    validation_masks_path = data_path + validation_folder_masks + '/'

    print('.............................')
    print('Folder and files for validation images and masks:')
    print('Images:', validation_images_path)
    print(validation_images)
    print('Masks:', validation_masks_path)
    print(validation_mask)

    # Go through validation images and run them through the network
    print('.............................')
    print('Predicting the validation images ...')
    for i in range(len(validation_images)):
        x = validation_images[i]
        y = validation_mask[i]

        name = x    # Name for result file

        # Read images
        path = validation_images_path + x
        x = cv2.imread(path, cv2.IMREAD_GRAYSCALE)
        #x = cv2.resize(x, (img_W, img_H))   # Resize if necessary
        x = x/255.0 # Normalize data
        x = np.expand_dims(x, axis=-1)  # Expand dimension to (img_W,img_H,1)
        x = x.astype(np.float32)    # Turn into np.array float32

        # Read mask
        path = validation_masks_path + y
        y = cv2.imread(path, cv2.IMREAD_GRAYSCALE)
        #y = cv2.resize(y, (img_W, img_H))      # Resize if necessary
        # The masks are in values (0,1,2)

        # Expand dimension to (img_W, img_H, 1) (this time 1, just for plotting)
        y = np.expand_dims(y, axis=-1)
```

```
        # Adjust values for plotting
        y = y*(255/(numberClasses-1))
        y = y.astype(np.float32)
        # if no padding - Pad image to get correct size.
        y = resize_with_crop_or_pad(y, img_W, img_H)
        print('Shape y: ', y.shape)

        # Prediction
        print('Predicting:', name)
        p = model.predict(np.expand_dims(x, axis=0))[0]
        # (1,512,512,3) with padding, and without padding (1,388,388,3)

        # Set the values such that the prediction can be plotted
        p2 = np.amax(p, axis=-1)     # This is to plot the certainty of chosen value
        p = np.argmax(p, axis=-1)

        # Plotting the certainty
        p2 = p2 * (255/(numberClasses-1))
        plt.figure()
        plt.imshow(p2)
        plt.show()
        cv2.imwrite(save_path + 'prediction' + name, p2)

        # Expanding dimensions to (img_W,img_H,1)
        p = np.expand_dims(p, axis=-1)
        p = p*(255/(numberClasses-1))    # Adjust values for plotting
        p = p.astype(np.float32)
        # Pad image to get correct size
        p = resize_with_crop_or_pad(p, img_W, img_H)
        print('Shape p: ', p.shape)
        #p = np.concatenate([p, p, p], axis=2)

        # FINAL IMAGE
        # Put the images all together
        x = x*255.0 # Go back to original values
        x = x.astype(np.int32)
        # Add a line between the images
        line = np.ones((img_H, 10, 1))*255
        final_image = np.concatenate([x, line, y, line, p], axis=1)

        fig = plt.figure()
        plt.imshow(final_image)
        plt.show()
        plt.close(fig)

        # SAVE the results image
        cv2.imwrite(save_path + name, final_image)



## ======================================================================================
#
#                                   RUN THE MAIN FUNCTION
#
## ======================================================================================

if __name__ == '__main__':
    main()
```
